# Successful treatment of corneal hypertrophic scar in Hurler syndrome

**DOI:** 10.1002/ccr3.9112

**Published:** 2024-06-24

**Authors:** Nima Koosha, Matin Irajpour, Zeynab Rostamiyan, Ali Shahsavari, Ali Forouhari, Mohsen Pourazizi

**Affiliations:** ^1^ Isfahan Eye Research Center, Department of Ophthalmology Isfahan University of Medical Sciences Isfahan Iran

**Keywords:** cornea, corneal scar, hurler syndrome, mucopolysaccharidosis, superficial keratectomy

## Abstract

In Hurler syndrome, corneal opacification is a common finding but rarely manifests as hypertrophic scars. A 6‐year‐old boy with Hurler syndrome had a hypertrophic scar on his left eye, which was successfully treated with superficial keratectomy.

## Graphical abstract

Hypertrophic scar on the cornea in patient with Hurler syndrome.

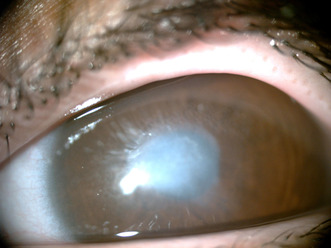

## 1 INTRODUCTION

Mucopolysaccharidosis type I (MPS‐I) is a genetic disorder characterized by progressive multisystem deterioration due to accumulation of dermatan and heparan sulfate that is, two major subclasses of glycosaminoglycans. This rare autosomal recessive inherited disease is caused by the deficient activity of the lysosomal hydrolase alpha‐L‐iduronidase (IUDA) which is involved in glycosaminoglycans metabolism. MPS I is further divided into severe (Hurler syndrome) and attenuated (Hurler‐Scheie syndrome/Scheie syndrome) phenotypes. Individuals affected by Hurler syndrome usually present initial manifestations during their first year of life. Dysfunction in multiple organs leads to various somatic and neurological signs and symptoms. Eventually if left untreated, cardiorespiratory failure can result in death within the first decade of life.^1^, ^2^

Ophthalmic issues are present in all the patients with severe MPS I.^1^ Corneal opacification (clouding), abnormal corneal hysteresis,^3^ glaucoma,^4^ retinal degeneration,^5^ optic nerve head edema and atrophy,^6^ decreased visual acuity and high hyperopia^7^ are the main ophthalmic manifestations among the patients.^1^, ^8^ In the following report we describe a 6‐year‐old boy with Hurler syndrome who presented with mild corneal clouding and a nodular lesion on the left cornea, which was successfully treated with superficial keratectomy (SK).

## 2 CASE HISTORY

A 6‐year‐old Iranian boy, confirmed to have MPS type I using a genetic study and with a positive family history of MPS I in his brother, was referred to our out‐patient clinic of ocular diseases of referral eye hospital of Isfahan University of Medical Sciences, Iran. The patient had been complaining about visual disturbance for the past 2 days. The patient had no history of any ocular trauma, mechanical or medical intervention and any ocular manipulation.

Extraocular manifestations of the patient were as follows: developmental delay, short stature, thoraco‐lumbar kyphosis, facial coarsening, enlarged lips, cardiomyopathy, and sensorineural hearing loss.

## 3 DIFFERENTIAL DIAGNOSIS, INVESTIGATIONS, AND TREATMENT

**FIGURE 1:**
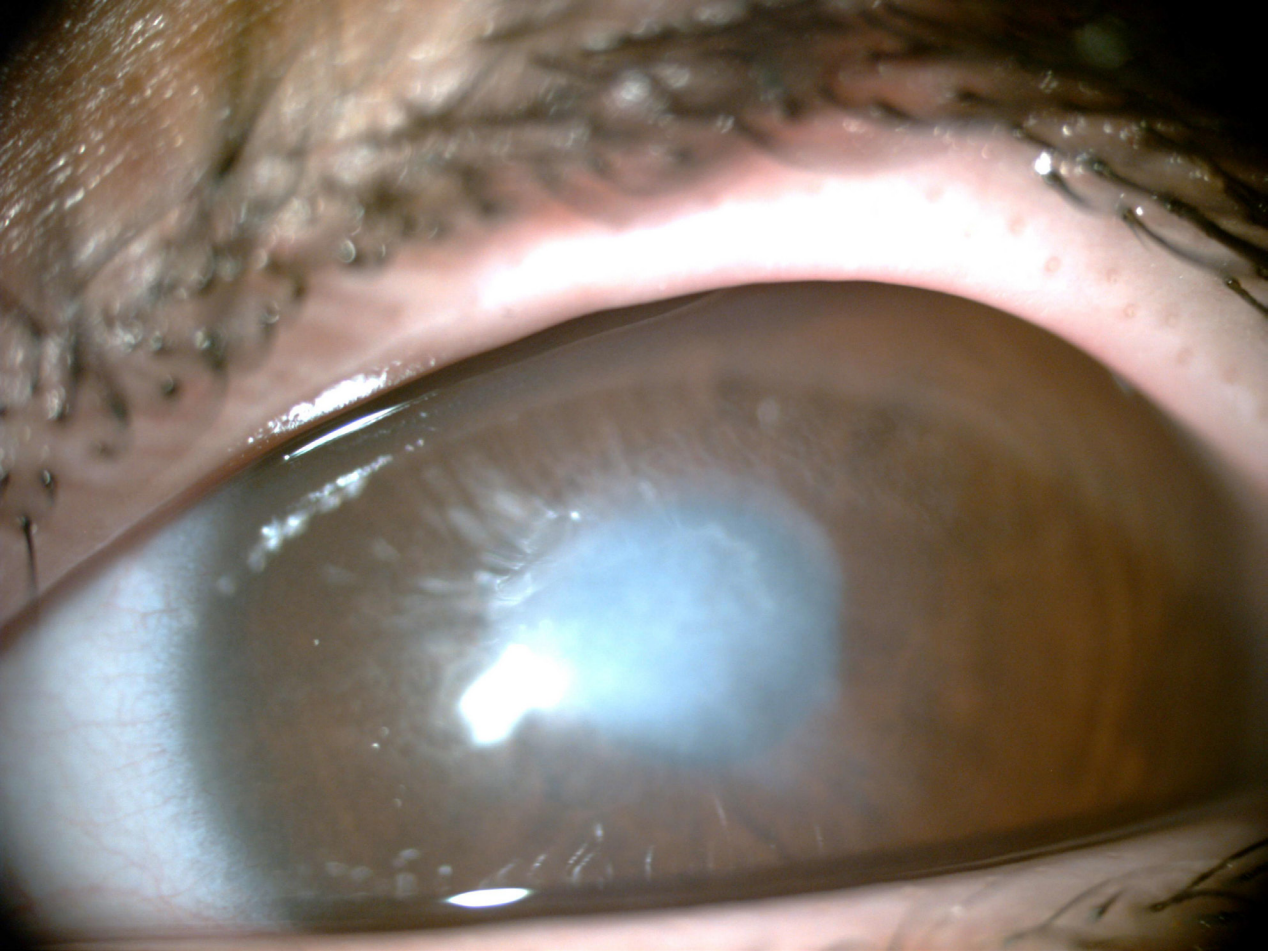
Mild corneal clouding with a hypertrophic scar on the left cornea.

**FIGURE 2:**
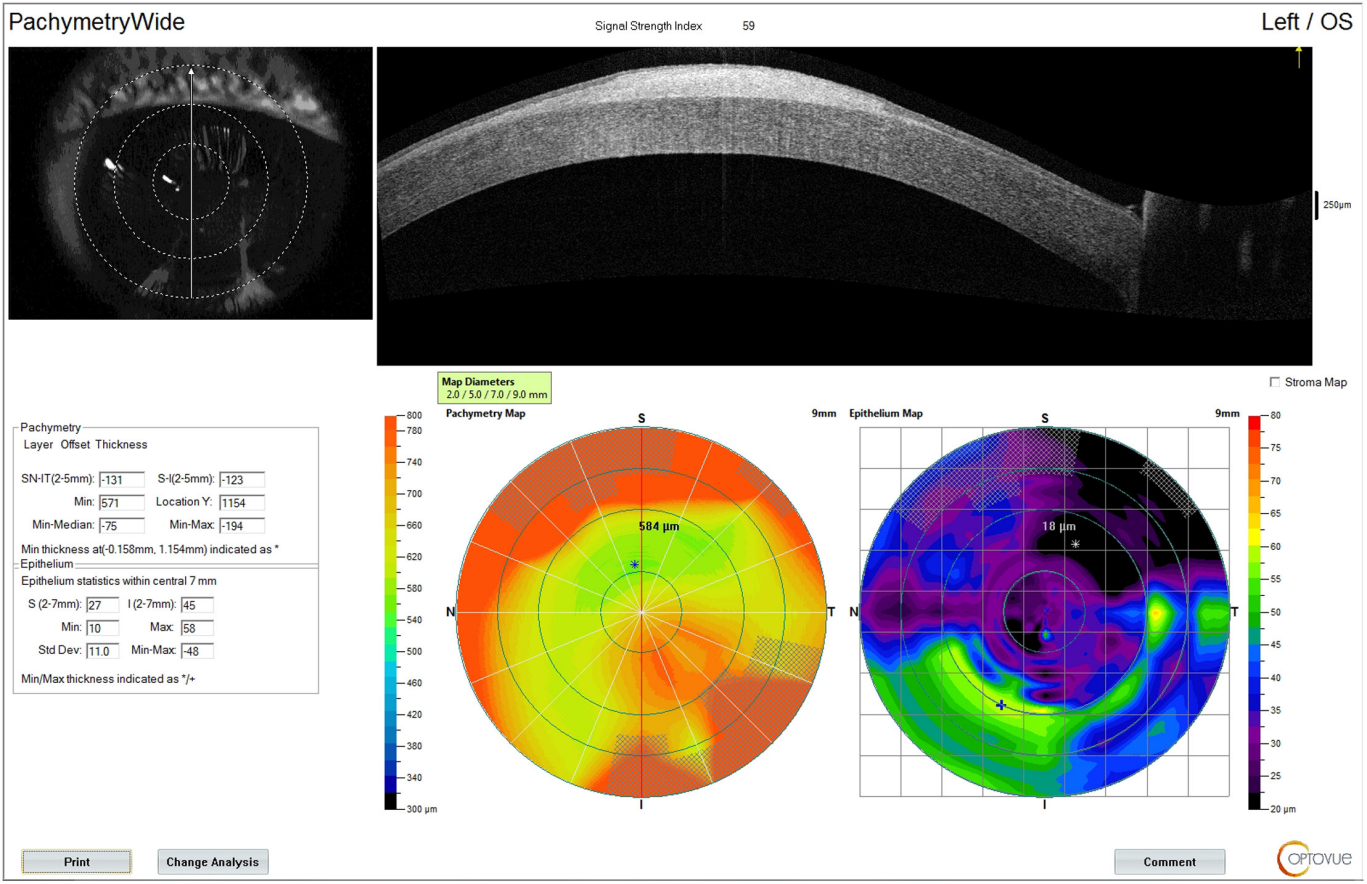
Hyperreflective lesion on anterior optical coherence tomography compatible with hypertrophic scar on the left central cornea.

On slit lam examination there was a mild corneal clouding in both sides and a hypertrophic scar on the central cornea of left eye (Figure 1). Dilated funduscopic examination was unremarkable. Diagnoses was established up by clinical examination and then confirmed using ophthalmic optical coherence tomography (OCT) (Figure 2).

Patient underwent SK and the postoperative course was uneventful without any further complications. After intravenous sedation, operated eye was anesthetized using Tetracaine ophthalmic drops (Anestocaine, Sina Darou Laboratories, Tehran, Iran). Corneal epithelium was removed following the instillation of alcohol drops (20% alcohol for 15 s). Hypertrophic and fibrous tissues were scraped using a #64 beaver blade. Mitomycin‐C (MMC 0.02%) soaked sponge was placed over the area of debrided cornea for 90 s. The cornea was irrigated with normal saline solution and a bandage contact lens (Aquvue Oasys) was placed on the eye. Postoperative medications regimen included the application of topical levofloxacin (Sina Darou, Tehran, Iran) and Betamethasone (Sina Darou, Tehran, Iran), both of which were administered four times a day for a duration of 1 month.

## 4 OUTCOME AND FOLLOW‐UP

**FIGURE 3:**
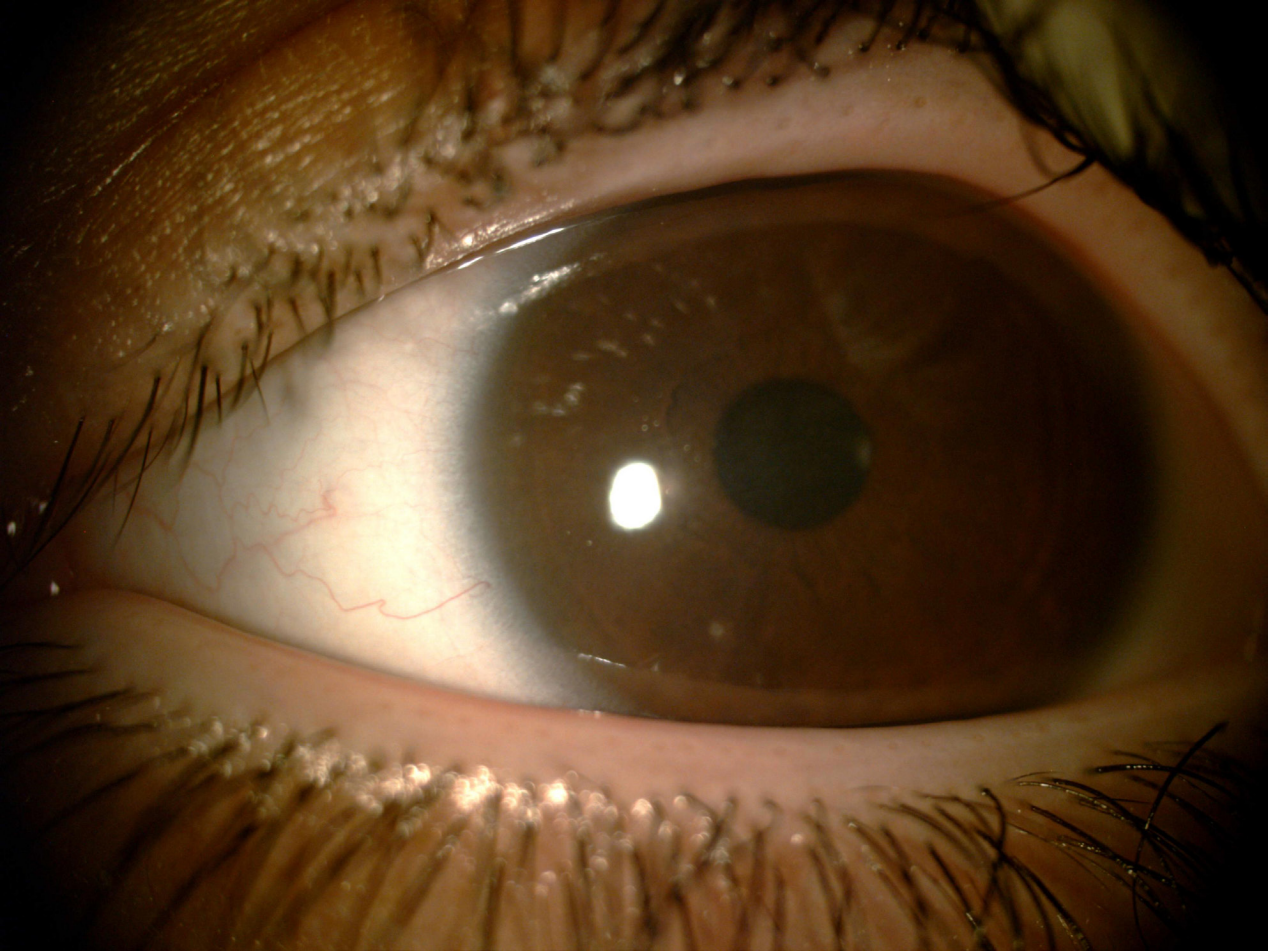
Normal thickness of the cornea after the operation.

No signs of inflammation were observed. After the operation, measured corneal thickness was within the normal range. Also, the patient was satisfied with their visual acuity and ability in 4 weeks follow‐up visit (Figure 3). During 12‐months of follow‐up, no evidence of recurrence was observed.

## 5 DISCUSSION

According to the global MPS I registry outcomes,^9^ 70.9% of Hurler cases will manifest corneal clouding at the median age of 1.1 years. The treatments mentioned were ineffective in prevention or reversing the corneal clouding in MPS patients.^10^, ^11^, ^12^, ^13^

Nodular lesions on the cornea are a rare occurrence and can have various etiologies.^14^ Corneal hypertrophic lesions, such as keloids, have been documented in several congenital syndromes and ophthalmic anomalies (e.g., Lowe's syndrome and Rubinstein‐Taybi syndrome); however, primary keloid lesions are seldom reported.^15^ As far as we are aware, this is the first reported case of a patient with Hurler syndrome and corneal nodular lesion in the literature.

SK was introduced in 1952^16^ and has been utilized to treat ocular surface disorders. In this simple procedure the corneal epithelium is debrided whilst the Bowman's layer is preserved.^17^ Typically, the manual dissection of superficial corneal layers is performed using a surgical blade or sterile sponge.^18^ The use of MMC, as an antimetabolite with the goal of diminishing the activity of keratocytes and fibroblasts, is recommended to prevent the recurrence of the lesion and decrease the likelihood of corneal haze following SK.^18^ SK is a very effective treatment method for corneal nodules and has is demonstrated to have remarkable efficacy in terms of recurrence prevention. However, it has some limitations such risk of postoperative corneal haze.^18^

In conclusion, we described the coincidence of a nodular hypertrophic corneal lesion and corneal clouding in a 6‐year‐old patient with Hurler syndrome, highlighting a rare but possible manifestation of this disease. Furthermore, we have presented SK as a successful treatment method of the specified lesion.

## AUTHOR CONTRIBUTIONS

**Nima Koosha:** Conceptualization; investigation; project administration. **Matin Irajpour:** Resources; writing – review and editing. **Zeynab Rostamiyan:** Resources; writing – original draft. **Ali Shahsavari:** Data curation; investigation; writing – original draft. **Ali Forouhari:** Data curation; resources; writing – original draft. **Mohsen Pourazizi:** Conceptualization; project administration; supervision; writing – review and editing.

## FUNDING INFORMATION

The authors received no financial support for the research, authorship, or publication of this article.

## CONFLICT OF INTEREST STATEMENT

Authors have no conflict of interest to disclose.

## ETHICS STATEMENTS

This case report was conducted in accordance with the ethical standards laid down in the 1964 Declaration of Helsinki and its later amendments.

## CONSENT

Written informed consent was obtained from the patient and their guardian. Details that might disclose the identity of the patient have been omitted.

## Data Availability

Anonymized data is accessible upon request from the corresponding authors.
